# Surgical resection of massive liposarcomas at the extremities: a 10-year experience in a referral musculoskeletal sarcoma unit

**DOI:** 10.1186/s12957-015-0622-6

**Published:** 2015-06-25

**Authors:** Bárbara Ortiz-Ibáñez, José V. Amaya, Francisco Baixauli, Manuel Angulo, Empar Mayordomo-Aranda, Carlos Barrios

**Affiliations:** Department of Orthopedic Surgery, Hospital de Sagunto, Valencia, Spain; Institute for Research on Musculoskeletal Disorders, Valencia Catholic University, Quevedo 2, 46001 Valencia, Spain; Musculoskeletal Sarcoma Unit, La Fe University and Technologic Hospital, Valencia, Spain; Department of Pathology, La Fe University and Technologic Hospital, Valencia, Spain

**Keywords:** Liposarcoma, Massive tumour size, Soft tissue sarcomas, Wide surgical excision

## Abstract

**Background:**

There is still no consensus regarding the management of patients with massive liposarcomas located in the extremities. Several discrepancies related to the aggressiveness of the surgery and the application of concomitant radiotherapy and chemotherapy treatments remain controversial. The purpose of this study was to analyse the clinicopathological characteristics, prognostic factors and outcomes of a series of patients with massive liposarcomas of the extremities who were treated at a referral hospital specializing in musculoskeletal oncology.

**Methods:**

This was an observational, descriptive and retrospective case series covering 10 years of clinical practice. The records of 26 adults, 14 men and 12 women, with localized massive liposarcomas in the extremities were studied. The average age was 53 years. The patients were treated from January 2003 until January 2012. Wide surgical resections with limb-sparing surgeries were performed for most patients (96.2 %).

**Results:**

The average tumour size was 15.1 ± 6.8 cm in the greatest dimension. Regarding the histological subtypes, there were 11 well-differentiated or atypical lipomatous tumours (42.3 %), 10 myxoid (38.5 %) and 5 (19.2 %) round cell and pleomorphic liposarcomas. Regarding the malignancy grades, 19 cases (73 %) were classified as low grade. Among these low-grade tumours predominated the well-differentiated subtype (57.9 %). Within high-grade tumours, the round cell and pleomorphic subtype was most frequent (57.1 %; *p* = 0.011). Radiotherapy was additionally applied to 12 patients (46.2 %) and adjuvant chemotherapy to 5 (19.3 %). Tumour recurrence was observed in only 2 cases (7.7 %). Only 1 of these cases developed lung metastatic dissemination.

**Conclusions:**

Across the entire series, these massive tumours did not compromise the survival of the patients. The histologic subtype and the malignancy degree were closely related. Proper and early diagnosis and therapeutic management of these patients via the application of wide-margin surgical excision are essential to ensure long-term survival.

## Background

Liposarcoma is one of the most common soft tissue sarcomas in adults, accounting for approximately 10 to 30 % of these tumours [1, 2]. Up to 80 % of liposarcomas involve the lower extremities, and these tumours are primarily deep seated in the proximal aspect of the thigh [3]. The risks of recurrence and metastatic dissemination have been related to the histologic malignancy grade and tumour size [3, 4].

Based on morphological and cytogenetic aberrations, liposarcomas are commonly classified into four subtypes: well-differentiated, dedifferentiated, myxoid and pleomorphic with round cells [5]. The well-differentiated and dedifferentiated subtypes represent 43 and 16 % of all liposarcomas, respectively, and are most frequently located in the retroperitoneum. The myxoid and the pleomorphic with round cell subtypes account for 29 and 12 % of liposarcomas, respectively, and most often affect the extremities [5]. The myxoid type exhibits an intermediate malignant behaviour and predominately occurs in adults and in the lower extremities, particularly the thighs, buttocks and the popliteal fossae. The round cell and pleomorphic variants are typically aggressive and often develop metastatic dissemination [6–9]. Most cases appear in adults over the age of 50 years and without a sex predilection [6, 8–10].

All subtypes are associated with a high risk of local recurrence unless they are properly excised [11]. The well-differentiated subtype can be separated from the other four types because of its lowest recurrence rate and much better prognosis [8]. The recurrence rates range from 5 to 83 % depending on the histologic subtype and location [5]. Regarding prognostic factors, both pathology and tumour size are extremely valuable. In a large series of 418 cases, the percentage of round cells and the tumour diameter were directly correlated with increased risks of metastases and death [4]. The importance of the histological subtype has been emphasized in a recent review in which the pathologic characteristics were found to be the main predictor of death from sarcoma [12]. Therefore, the four subgroups of liposarcoma exhibit different natures and patterns of behaviour [5, 11, 13, 14].

Patients with well-differentiated liposarcomas at the extremities usually present with a painless, slowly growing soft mass, which can be accurately delineated with magnetic resonance imaging (MRI) [15, 16]. The extremity lesions have the potential to hold locally aggressive behaviour, but with no metastatic potential unless they dedifferentiate [17]. Based on these different clinical behaviours, it has been proposed that the term atypical lipomatous tumour should be used instead of “well-differentiated liposarcoma”.

Clinical presentations with lung metastases, advanced age and large tumour sizes are associated with poor prognoses [18, 19]. However, regarding tumour size, appropriate wide surgical resections seem to decrease the recurrence rate to near 0 even for massive tumours [20]. A comparison of two clinical trials seeking to determine the prognostic factors for tumour progression revealed that lesions localized to the extremities are associated with favourable prognoses in young people, and adjuvant chemotherapy results in a greater relapse-free survival only in patients older than 30 [21].

The primary treatment for high-risk patients is surgical resection and local control with adjuvant radiotherapy [22–25]. The resection of local recurrences localized to the extremities can provide results that are similar to those of primary tumour resection. In cases of unresectable pulmonary metastases or extrapulmonary metastatic sarcomas, the prognoses are very unfavourable, and systemic chemotherapy is required. In these cases, surgery might be considered as a palliative treatment [5, 26, 27].

There is still no consensus regarding the management of patients with massive liposarcomas located in the extremities. Several discrepancies related to the aggressiveness of the surgery and the application of concomitant radiotherapy and chemotherapy treatments remain controversial.

Initially, patients with well-differentiated liposarcomas in the extremities are expected to have a better chance of being cured; therefore, wide excision should be the initial therapy of choice. Even in cases with lesions in contact with major nerves or blood vessels, conservative resection preserving these critical structures and the limb function has proved to be highly effective in terms of low recurrence and metastatic spreading [17].

Given this knowledge background, the current study aimed to analyse a series of patients with massive liposarcomas of the extremities who were treated at a referral tertiary hospital with a specialized musculoskeletal oncologic unit. The clinicopathological characteristics, the prognostic factors and the outcomes were reviewed. To our knowledge, this study is innovative given the limited literature that has been published regarding massive liposarcomas.

## Methods

This was a retrospective, observational and descriptive study of a series of cases. We analysed a total of 26 patients, with a mean age of 53 years (15.6 SD), who were diagnosed with liposarcoma in the extremities. Patients’ ages ranged from 27 to 86 years. Of the 26 patients studied, there were 14 males (mean age 56.8 ± 17.5 SD) and 12 females (mean age 48.5 ± 12.3 SD). The time of follow-up was calculated from the date of the surgical treatment. The median follow-up period was 38.5 months (13.5 SD).

This study was based on data from the medical records that were registered from January 2003 to December 2012. Data from patients who received any of the following types of treatment were collected: limb-saving surgery (25 cases), radical surgery with amputation (1 case), adjuvant radiotherapy (8 patients), adjuvant chemotherapy (1 case) and combined radiotherapy with chemotherapy (4 cases). All patients were treated in a musculoskeletal oncology unit of a tertiary referral hospital. Written informed consent was obtained from the patients for the publication of this report.

Data regarding age, sex, histological diagnosis, malignancy grade, topographic location, tumour size, clinical comorbidities, metastasis, surgical treatment, concomitant therapy, local recurrence and clinical situation were collected from the medical records.

Regarding the clinical symptoms, most of the patients presented with a palpable mass; however, in a single patient, the tumour was incidentally discovered in the context of an iliac ischemic process. At the time of patients’ referral, 20 presented with primary lesions and six patients had been previously operated and were referred with recurrent masses. The main comorbidities were several cardiovascular risk factors.

The predominant tumour location was the thigh (20 patients). The other 6 patients presented with tumours in different locations: 2 were in the arm, 2 were in the upper posterior thigh involving the posterior pelvic muscles and 2 were in the knee.

Across the entire series, 19 cases (73 %) were labelled as low-grade malignancies (19 cases, grade I). Among the remaining 7 cases with high-grade tumours, 5 were labelled as grade II and 2 were labelled as grade III.

The statistical analyses of the data were initially performed with descriptive calculations of frequencies and percentages for the qualitative variables. The means, standard deviations and minimum and maximum values were also obtained for the quantitative measurements. These analyses were conducted across the whole sample. Patients were also segregated in subsamples by sex, histologic subtype and tumour size. Fisher’s exact tests with contingency tables were applied to assess differences between groups. Differences were considered statistically significant when *p* < 0.05.

## Results

According to age and grade, the patient distribution consisted of 9 low-grade and 4 high-grade cases in patients below the age of 50 years. Among the patients older than 50 years, there were 10 low-grade and 3 high-grade cases. Regarding the histological diagnoses, there was a clear tendency toward specific histological types according to sex; the well-differentiated type predominated in males (57.1 %), and the myxoid form predominated in the women (58.3 %; Table 1).

**Table 1:** Histologic characteristics and locations of the liposarcomas according to gender

	Males	Females
	*n* (%)	*n* (%)
Histologic type		
Well-differentiated	8 (57, 1)	3 (25)
Myxoid	3 (21, 4)	7 (58, 3)
Round cell and pleomorphic cells	3 (21, 4)	2 (16, 7)
Grade		
High-grade	10 (71, 4)	9 (75)
Low-grade	4 (28, 6)	3 (25)
Location		
Arm	1 (7, 1)	1 (8, 3)
Thigh	10 (71, 4)	10 (83, 3)
Upper posterior thigh and gluteal muscles	1 (7, 1)	1 (8, 3)
Knee	2 (14, 3)	–

The percentages in parentheses indicate the proportion of cases within the category. There were no differences between males and females in the histologic characteristics and tumour location according to Fisher’s exact test

**Fig. 1:**
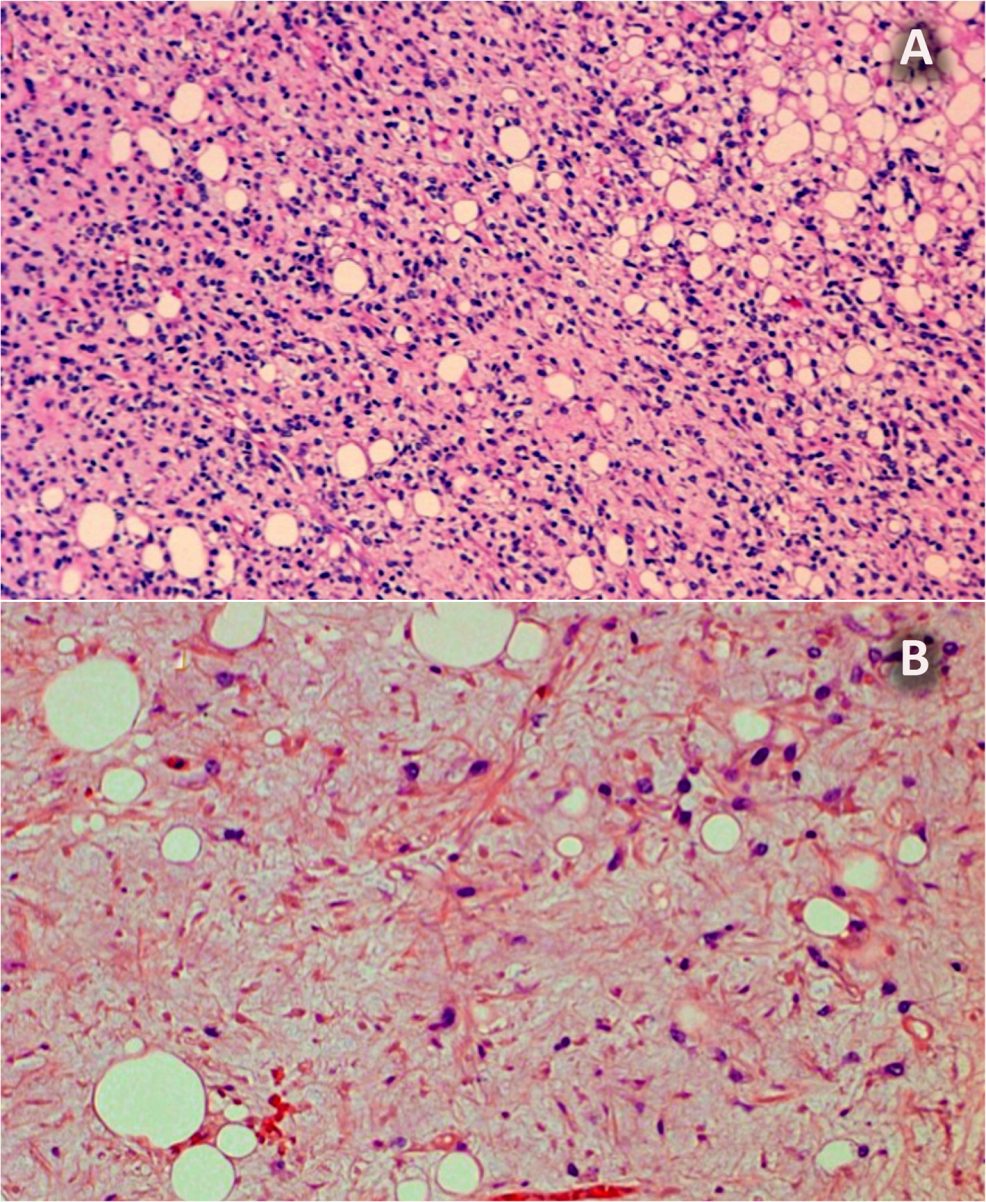
Myxoid liposarcoma. **a** Abundant small cells, very few mature adipocytes and necrotic areas. Haematoxylin-eosin (HE) 30 μm. **b** Detail of a rather myxoid area, with scattered atypical adipocytes, lipoblasts and small round cells in a bluish myxoid background with typical delicate neoformed vessels (HE, 60 μm)

**Fig. 2:**
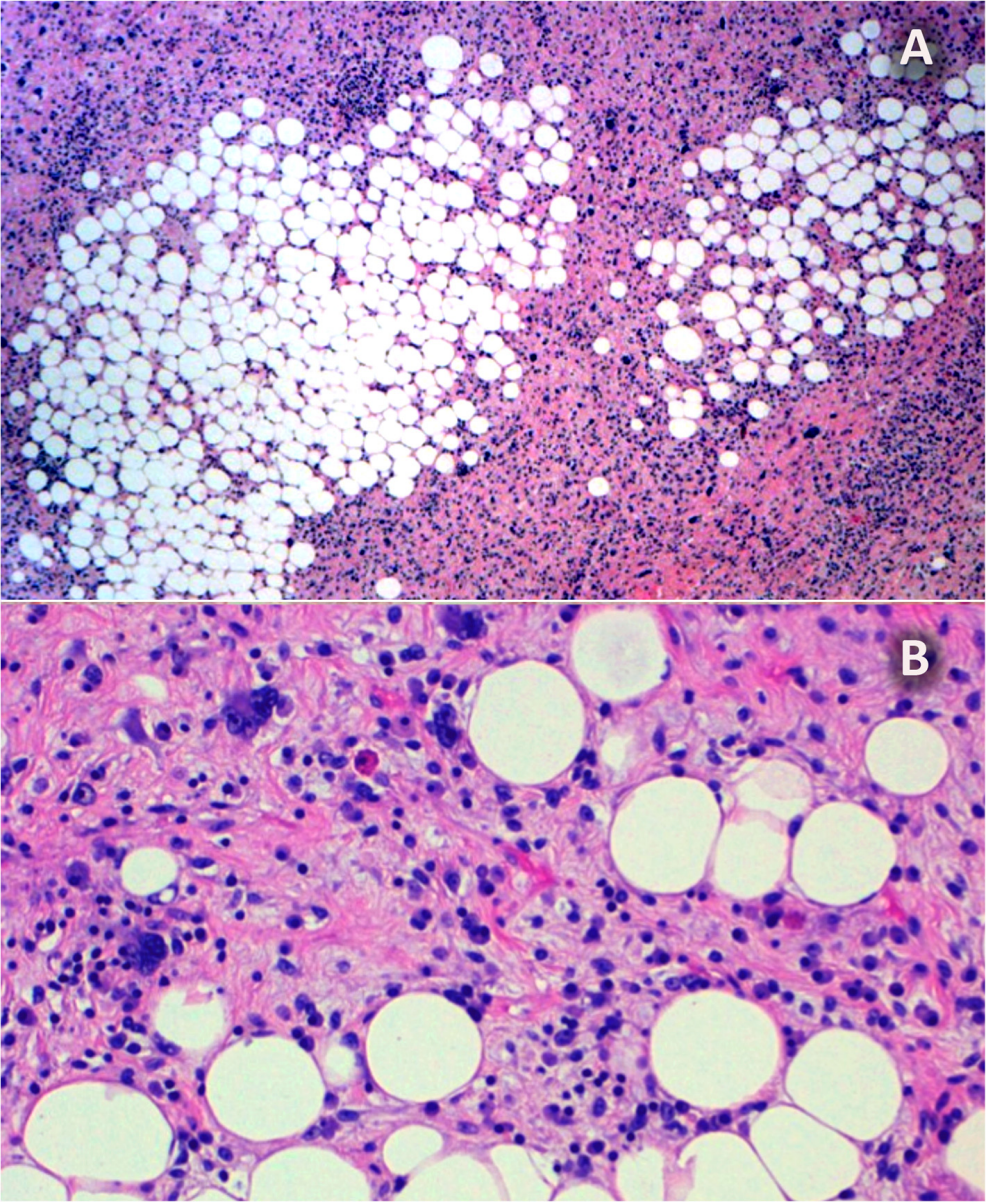
Dedifferentiated liposarcoma. **a** Dense highly pleomorphic sarcomatoid areas intermixed with low-grade liposarcoma adipose subtype (HE, 12 μm). **b** Areas of low-grade liposarcoma, adipose subtype, with atypical adipocytes in fibrous septa intermixed with mature adipocytes and lipoblasts (HE, 60 μm)

Among the low-grade tumours, the well-differentiated histologic subtype was most common (11 of 19 cases); among the high-grade tumours, the predominant histological type was the round and/or pleomorphic cell type (4 of 7 cases; *p* = 0.011). The well-differentiated histological type was never classified as high grade. The myxoid type exhibited a similar ratio of cases in both groups (Table 2). In some cases, various liposarcoma subtypes were mixed; these cases were classified according the most dominant variant (Figs. 1 and 2).

**Table 2:** Age, histologic subtype, location and metastatic development according to the degree of malignancy

	Low-grade	High-grade
	*n* (%)	*n* (%)
Age (years)		
≤50	9 (47, 4)	4 (57, 1)
>50	10 (52, 6)	3 (42, 9)
Histologic type		
Well-differentiated	11 (57, 9)	–
Myxoid	7 (36, 8)	3 (42, 9)
Round and/or pleomorphic cells	1 (5, 3)	4 (57, 1)
		*p <* 0.05
Location		
Arm	–	2 (28, 6)
Thigh	15 (78, 9)	5 (71, 4)
Upper posterior thigh and gluteal muscles	2 (10, 5)	–
Knee	2 (10, 5)	–
Metastases		
M1	–	1 (14, 3)
M0	19 (100)	6 (85, 7)

The percentages in parentheses indicate the proportion of cases within the category. Fisher’s exact test was used for statistical analysis

**Fig. 3:**
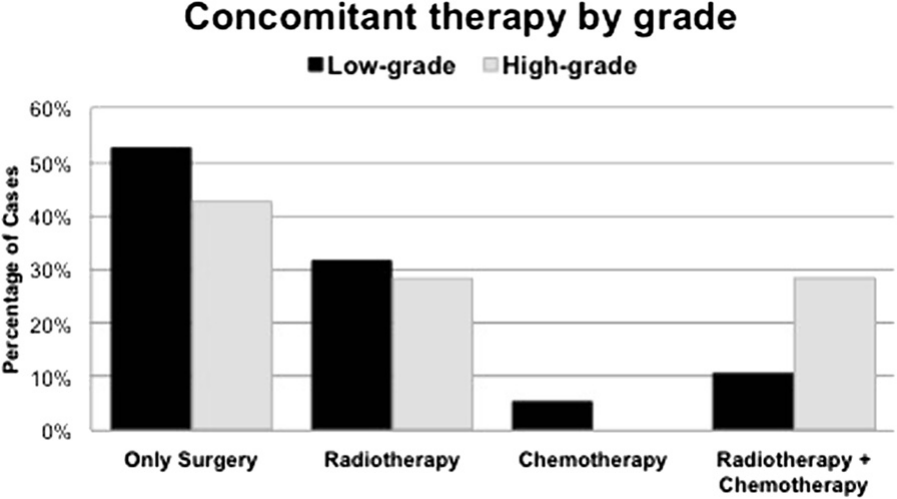
Distribution of concomitant therapy according to the degree of malignancy

The surgical treatments consisted of wide resections of the tumours with limb-preserving techniques in 25 cases (96.2 %; Fig. 3). All lesions involving or adjacent to major nerves or blood vessels were marginally resected with careful dissection of these critical structures. The sciatic nerve and the femoral and the popliteal arteries were preserved in all patients with lower-extremity lesions. In the two patients with upper-arm lesions, the mean peripheral nerve trunk nerves and the major arteries (brachial and radial) were also preserved.

**Fig. 4:**
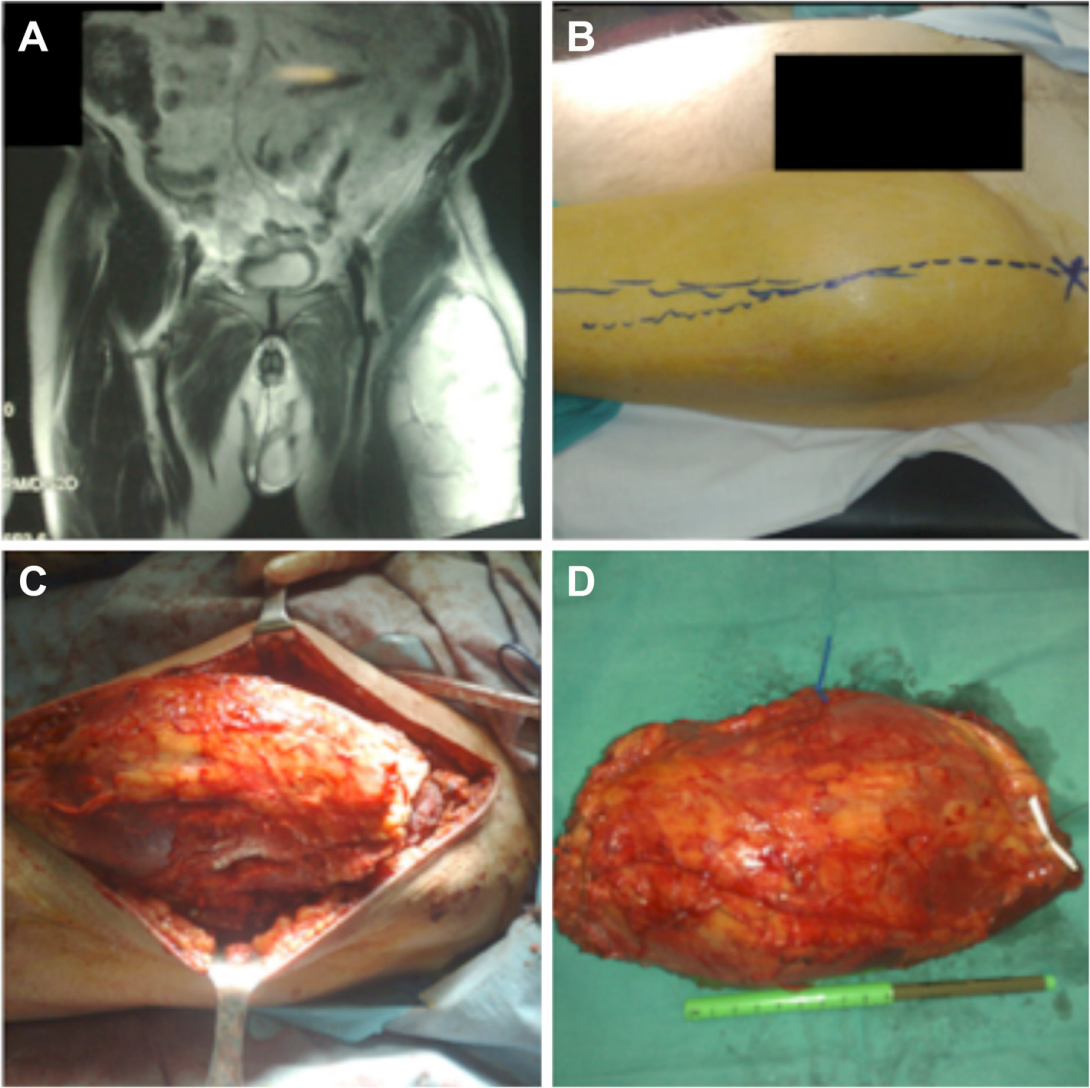
A 69-year-old male with myxoid liposarcoma grade II located in the rectus muscle of the left thigh. **a** Preoperative MRI, December 2008; **b** clinical appearance; **c** intraoperative aspect during surgical resection of the tumour; and **d** excised tumour part measuring 22 × 15 cm. This patient received adjuvant radiotherapy

Only 1 case required amputation of the limb (3.8 %); this patient was diagnosed in the context of an iliac ischemia. The distribution of concomitant therapies according to the degree of tumour malignancy revealed some relevant features, i.e. 52.6 % of the low-grade tumours and 42.9 % of the high-grade did not receive any treatment other than surgery. Radiotherapy was added to surgery for 31.6 % of the low-grade and 28.6 % of the high-grade tumours (Figs. 3 and 4). Chemotherapy as a single associated treatment was administered only to a single low-grade case. Finally, the combination of radiotherapy and chemotherapy was applied to the same number of patients in each group according to the degree of malignancy (Table 3).

**Table 3:** Applied treatments and clinical evolution according to gender

	Total	Males	Females
	*n* (%)	*n* (%)	*n* (%)
Concomitant therapy			
Only surgical excision	13 (50)	8 (57, 1)	5 (41, 7)
Radiotherapy	8 (30, 8)	4 (28, 6)	4 (33, 3)
Chemotherapy	1 (3, 8)	–	1 (8, 3)
Radiotherapy + chemotherapy	4 (15, 4)	2 (14, 3)	2 (16, 7)
Local recurrence			
Yes	2 (7, 7)	–	2 (16, 7)
No	24 (92, 3)	14 (100)	10 (83, 3)
Metastases			
M1	1 (3, 8)	–	1 (8, 3)
M0	25 (96, 2)	14 (100)	11 (91, 7)

The percentages in parentheses indicate the proportion of cases within the category. There were no differences between males and females in the applied therapy and in the clinical course according to the Fisher’s exact test

**Fig. 5:**
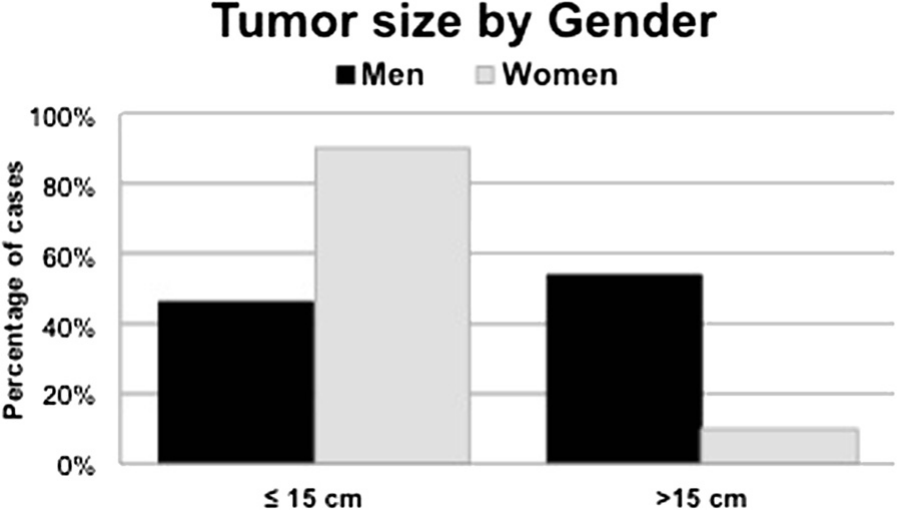
Tumour size by gender segregated by tumour sizes ≤15 and >15 cm in average maximum diameter

Across the entire series, the mean tumour size as measured following the surgical removal of the specimens was 15.1 ± 6.8 cm in the greatest diameter. Comparison of the sizes of the tumours across gender revealed that the tumour sizes of the men were slightly larger than those of the women (16.0 ± 7.3 in men and 14.2 ± 6.3 cm in women), but this difference was not significant due to the large dispersion of results, particularly in the men. However, the proportion of women who exhibited small tumours (≤15 cm) was 75 % (9 of the 12 women), while only 1 woman had a large tumour (8.3 %; *p* = 0.074; Fig. 5).

**Fig. 6:**
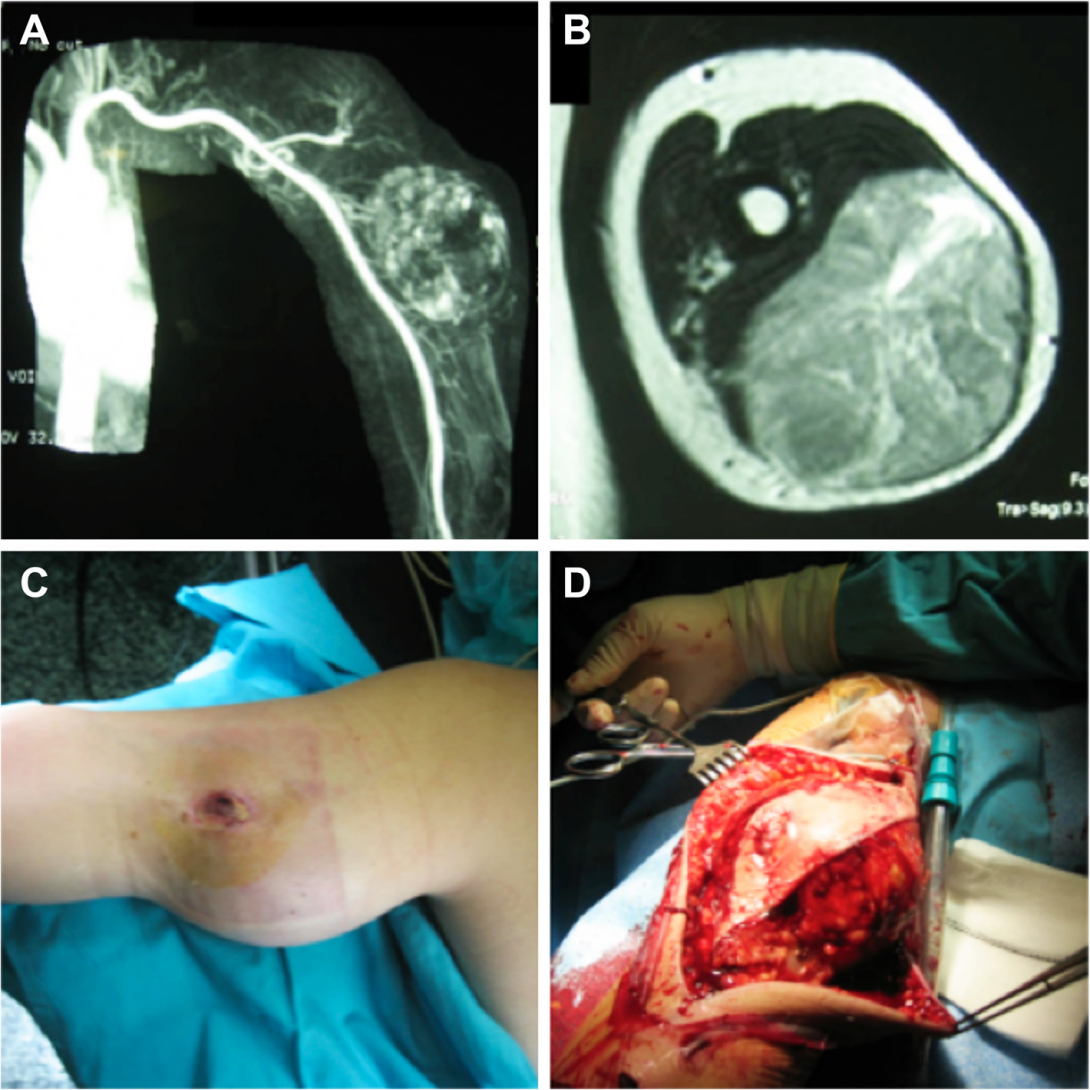
A 53-year-old female with grade III round cell pleomorphic liposarcoma (highly malignant) located in the triceps muscle of the left upper arm. **a** Resonance angiography showing an increase in tumour vascularization; **b** preoperative MRI, January 2007; **c** clinical appearance of the palpable mass; and **d** intraoperative appearance. The tumour measured 15 × 9 × 8 cm. This patient developed lung metastases and died 9 months after the diagnosis

At the last follow-up, 24 patients continued to be disease free without any loss of limb function. There were only two local recurrences, and both occurred in women younger than 50. Only 1 patient presented with metastases that were located in the lungs. This woman had a high-grade pleomorphic and round cell liposarcoma located in the upper arm. This was the case in the study that evolved to death, which occurred 9 months after the diagnosis (Fig. 6). The association between the development of metastasis and the evolution to death was statistically significant (*p* = 0.038). According to these data, the final local control rate was 92.3 %.

**Fig. 7:**
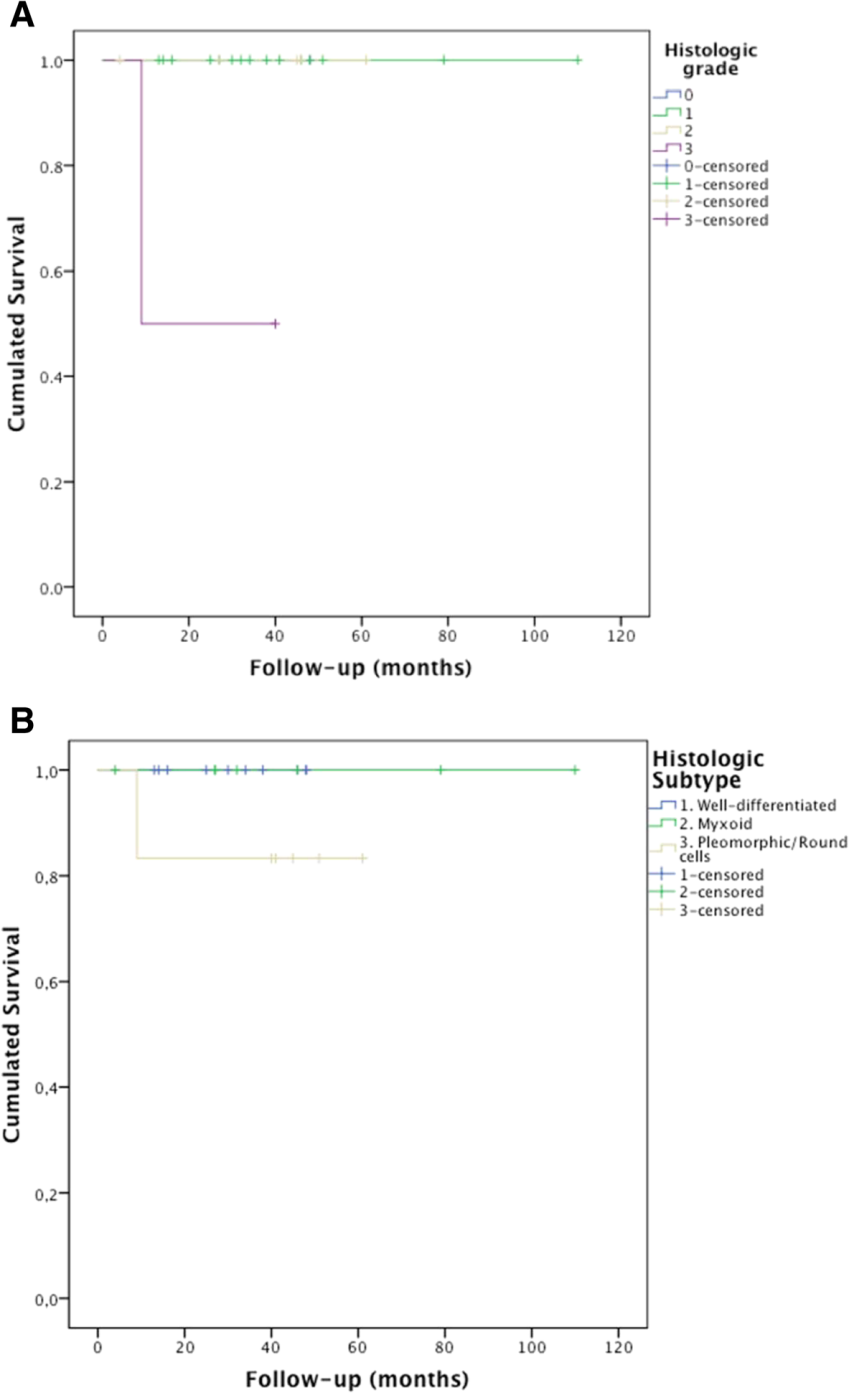
Survival (months) in relation to the malignancy grade (**a**) and the histological subtype (**b**) (January 2003–December 2012)

One of the two grade III cases died due to the tumour; thus, the survival in this group was 50 % (Fig. 7a). One of the four cases with a pleomorphic variant progressed to death; thus, the survival curves segregated by histological type revealed that the survival in these cases was 75 % (Fig. 7b).

## Discussion

The strength of this study lies in the inclusion of a series of patients with massive lower-extremity liposarcomas from a referral musculoskeletal oncology unit of a tertiary hospital. The sample might be therefore representative of liposarcomas with large clinical effects. Both the age and sex distributions matched with those in the literature related to smaller liposarcomas [5, 8].

In this series, the well-differentiated histological pattern was the most common in men and the myxoid form was the most common in women, in accordance with the results of previous studies [5, 8, 28, 29]. Regarding the degrees of tumour malignancies, low-grade neoplasms predominated among the well-differentiated type. Among the high-grade tumours, the round and/or pleomorphic cell type was the most common. The prevalence of the myxoid type was similar in the low- and high-grade groups. In this series, low-grade massive liposarcomas were found to be more than twice as frequent as their highly malignant counterparts.

In the current review, the tumours were predominantly localized to the proximal portion of the thigh, which agrees with previous studies [3, 12]. Because only these tumours were examined in the current study, the proportions of retroperitoneal tumours and other liposarcoma areas are unknown. The clinical presentation was a palpable mass in all cases. No pain was present upon presentation, but pain has been reported to be the first symptom in one third of cases in the literature [5, 30].

The differences in various clinical behaviours have generated confusion about the best way to clinically treat soft tissue sarcomas. Surgical tumour resection has been proposed to be the most effective treatment for soft tissue sarcomas [8, 11, 25]. Regarding liposarcomas, complete surgical excision in association with radiation offers the best local control [31, 32]. This therapeutic approach has been specifically proposed as the treatment of choice for the well-differentiated and myxoid subtypes [33]. The local recurrence rates reported in the literature only about extremity lesions range from 8 to 52 % [34–36]. In our series, the local recurrence rate was only 7.7 %, which is below the lower range of those reported previously.

In the current study focussed on massive liposarcoma at the extremities, all of the patients underwent wide resections of the tumours. In half of the cases, no additional treatments were applied. When the distribution of concomitant treatments was analysed according to the degree of malignancy of the tumour, some findings that deserved comment were revealed. As in other large series of soft tissue liposarcomas, radiotherapy was applied to one third of the patients with low-grade tumours and applied to a smaller proportion of the high-grade cases [3].

In a study conducted by Zagars et al. [33], the indication for chemotherapeutic therapy was a tumour size >5 cm. It has also been reported that myxoid liposarcomas exhibit good responses to chemotherapy [28]. However, according to the currently available literature, it is reasonable to include chemotherapy as a part of the treatment strategy for these tumours only in patients with untreated, advanced unresectable disease and patients with metastatic liposarcomas. In our series, chemotherapy was applied to 5 cases, 2 of which were low-grade cases and 3 were high-grade tumours. One of these 5 cases was a well-differentiated liposarcoma, 1 was a pure myxoid tumour, 2 were myxoid tumours with round cells and 1 was a pleomorphic liposarcoma. The scheme of surgery plus radiotherapy and/or chemotherapy was applied to the same number of patients in each group depending on the degree of malignancy. These data indicate that the addition of neoadjuvant chemotherapy or radiotherapy in this series did not follow the current recommendations for soft tissue sarcomas [21].

Local recurrence has been strongly associated with metastatic disease in soft tissue sarcomas [37]. In this study, local recurrence was only observed in two patients after being treated in the orthopaedic oncologic unit. However, a total of 6 patients underwent a revision surgery due to insufficient surgical margins in the initial surgery, performed outside the orthopaedic oncologic unit in 5 of theses cases. These findings are inconsistent with the proposals of other authors [18, 21] in terms of prognostic factors. The two cases with local recurrence did not meet the accepted criteria for bad prognoses; both of these cases were under 50 years old and appeared as de novo liposarcomas, and the histological subtypes were myxoid. In the current series, surgical resection of local recurrences of massive liposarcomas in the extremities produced results that were similar to those of the resection of the primary tumours because both of these patients are currently alive. The promising results of this series cannot be related to the high number of well-differentiated liposarcomas treated, because only 11 out of 26 patients (42 %) were identified.

In the current series, the average tumour size exceeded 15 cm; thus, the sample was divided into two groups based on whether the tumour was larger or smaller than 15 cm. Traditionally, the size limit used to discriminate large and small tumours has been 10 cm [3, 5]. This series exclusively included patients from a referral hospital. Therefore, the great majority of the tumours were massive and advanced in their evolutions. In our system, small liposarcomas have previously been treated in county primary hospitals. Therefore, large tumour size per se could not be analysed as a prognostic factor.

Only 1 patient with a high-grade pleomorphic round cell liposarcoma developed metastases that were located in the lungs. This patient was the only case in the study who exhibited an unfavourable evolution that led to death due to tumour spread. This case confirms the reported experience that has described lower survivorship of the pleomorphic variant and the presence of distant metastases as the major cause of death in patients with liposarcomas [29, 33]. Although a history of prior local recurrence and a positive resection margin significantly affected the prognoses of the patients, in the series of Zagras et al. [33], histologic subtype was the most important factor.

The study has some obvious limitations due to the nature of clinical reviews. Patients under 18 years of age were not included; therefore, the results cannot be extrapolated to adolescent and paediatric patients. However, the prevalence of liposarcomas in patients below the age of 18 is very small [1, 38]; thus, this limitation will affect only a small fraction of cases. Furthermore, the retrospective nature of this study implies that, in some cases, not all of the analysed parameters were included in the patient medical records. Although this information is systematically computerized in our institution, some of the oldest cases lacked surgery-related information and were consequently eliminated from the study. Another limitation is that the majority of the patients were referred from other hospitals, and some of them underwent previous operations. This feature complicated the collection of the clinical characteristics of the primary tumours from the medical records.

## Conclusions

In conclusion, wide excision surgery produces excellent results in the treatment of massive liposarcomas of the extremities in terms of not only low recurrence rate but also good limb function. Only 1 patient died of metastases. The results from series were highly satisfactory, although improvements in the criteria for the application of coadjuvant chemotherapy and radiotherapy would be desirable in the future.
